# Integron Digestive Carriage in Human and Cattle: A “One Health” Cultivation-Independent Approach

**DOI:** 10.3389/fmicb.2017.01891

**Published:** 2017-09-27

**Authors:** Delphine Chainier, Olivier Barraud, Geoffrey Masson, Elodie Couve-Deacon, Bruno François, Claude-Yves Couquet, Marie-Cécile Ploy

**Affiliations:** ^1^INSERM, CHU Limoges, UMR 1092, Université Limoges, Limoges, France; ^2^INSERM, CIC1435, CHU Limoges, Limoges, France; ^3^Laboratoire Départemental d’Analyses et de Recherches de la Haute-Vienne, Limoges, France

**Keywords:** integrons, digestive carriage, antimicrobial resistance, humans, bovine, One Health

## Abstract

**Objectives:** Dissemination of antimicrobial resistance (AMR) is a global issue that requires the adoption of a “One-Health” approach promoting integration of human and animal health. Besides culture-dependent techniques frequently used for AMR surveillance, cultivation-independent methods can give additional insights into the diversity and reservoir of AMR genetic determinants. Integrons are molecular markers that can provide overall and reliable estimation of AMR dissemination. In this study, considering the “One-Health” approach, we have analyzed the integron digestive carriage from stools of humans and cattle living in a same area and exposed to different antibiotic selection pressures.

**Methods:** Three collections of human [general population (GP) and intensive care unit patients (ICUs)] and bovine (BOV) stool samples were analyzed. The three main classes of integrons were detected using a multiplex qPCR both from total DNA extracted from stools, and from Gram-negative bacteria obtained by culture after an enrichment step.

**Results:** With the cultivation-independent approach, integron carriage was 43.8, 52.7, and 65.6% for GP, ICU, and BOV respectively, percentages being at least twofold higher to those obtained with the cultivation-dependent approach. Class 1 integrons were the most prevalent; class 2 integrons seemed more associated to cattle than to humans; no class 3 integron was detected. The integron carriage was not significantly different between GP and ICU populations according to the antibiotic consumption, whatever the approach.

**Conclusion:** The cultivation-independent approach constitutes a complementary exploratory method to investigate the integron digestive carriage of humans and bovines, notably within subjects under antibiotic treatment. The high frequency of carriage of integrons in the gut is of clinical significance, integrons being able to easily acquire and exchange resistant genes under antibiotic selective pressure and so leading to the dissemination of resistant bacteria.

## Introduction

Antimicrobial resistance (AMR) has increased to a dramatic extend and is a public health threat (Roca et al., 2015). This is particular true for Gram-negative bacteria (GNB) with the expansion of extended-spectrum beta-lactamase (ESBL) or carbapenemase-producing Enterobacteriaceae and with the emergence of new resistance mechanisms, as plasmid-mediated colistin resistance (Meletis, 2016). AMR is a global issue encompassing human, animal, and the environment. Efforts to tackle AMR dissemination thus require the adoption of a “One-Health” approach that promotes integration of public health and veterinary disease, food, and environmental surveillance (Sikkema and Koopmans, 2016). Culture-dependent techniques are frequently used for AMR surveillance in GNB. However, given the role of horizontal gene transfer in AMR dissemination, cultivation-independent methods give additional insight into the diversity and reservoir of AMR determinants.

Several studies indicated that integrons are molecular markers that can provide overall and reliable estimation of AMR dissemination (Amos et al., 2015; Gillings et al., 2015). Integrons are bacterial genetic elements capable of promoting the acquisition and expression of antibiotic resistance genes embedded within gene cassettes (Cambray et al., 2010). Three main classes of integrons are involved in the spread of AMR and several studies have underlined the link between integrons and multidrug resistance in GNB (Leverstein-van Hall et al., 2003; Barraud et al., 2014). In humans and animals, class 1 integrons are the most prevalent, followed by class 2 integrons (Barlow et al., 2004; Cocchi et al., 2007; Vinue et al., 2008; Barraud et al., 2014), whereas very few class 3 integrons are found, this class being mostly associated with environmental samples (Simo Tchuinte et al., 2016).

Nowadays, it is impossible to separate the animal and human resistance issues as genetic transfers exist between these two entities. In this study, considering a “One-Health” approach and the integron biomarker, we have taken benefit from a large collection of stools to analyze the digestive carriage of integrons in humans and bovines, in a cattle-rearing area. This study highlights the added-value of using two complementary approaches for detecting integrons, a cultivation-independent one performed directly from stools and a cultivation-dependent one from GNB and emphasizes the importance of the gut as a reservoir of genetic elements encoding antibiotic resistance.

## Materials and Methods

### Stool Samples

A collection of 567 stool samples from human and bovines has been previously established by our team. These samples were consecutively collected from 2006 to 2010 and stored at -80°C (human collection number DC-2008-604). All subjects originated from the same cattle-rearing area located in the southwestern France (Haute-Vienne, 5.520 km^2^). The stools were obtained from (i) subjects from the general population working in non-clinical units of Limoges hospital and attending industrial medicine clinics (‘GP,’ *n* = 194), (ii) patients hospitalized in the intensive care unit of the hospital (‘ICU,’ *n* = 245), (iii) bovines, mainly cows, from 55 different farms (‘BOV,’ *n* = 128). Only one stool sample per subject was collected. For the two human stool collections, on-going antibiotic therapies and previous antibiotic therapies during the last 3 months were reported; for bovines, no data regarding antibiotic consumptions were available.

### Bacterial Culture

Two hundred milligrams of stools were inoculated into 5 ml of Brain Heart Infusion broth (AES Laboratory, France) at 37°C overnight without agitation. The day after, 10 μl were inoculated onto Drigalski agar plates (bioMérieux, France) incubated for 18–24 h at 37°C. GNB with different morphotypes were selected for identification (ID) and antibiotic susceptibility testing (AST). ID was performed using the Vitek 2 system (Vitek2-ID-GNB, bioMérieux, France). AST was determined using the disk diffusion method according to the recommendations of the French Society for Microbiology with the 21 following drugs: amoxicillin (AMX), amoxicillin clavulanic acid (AMC), ticarcillin (TIC), ticarcillin clavulanic acid (TCC), cefotaxim (CTX) ceftazidim (CAZ), imipenem (IPM), kanamycin (K), gentamicin (GM), tobramycin (TM), amikacin (AN), streptomycin (S), spectinomycin (SPT), nalidixic acid (NA), pefloxacin (PEF), ciprofloxacin (CIP), sulfamethoxazole (SSS), trimethoprim (TMP), trimethoprim-sulfamethoxazole (SXT), chloramphenicol (C), and tetracycline (TET). Results categorized intermediate were reported as resistant in the results section.

### Integron Detection

Total DNA was extracted from 200 mg of stool samples using the QIAamp DNA stool mini kit (Qiagen, Courtaboeuf, France) following manufacturer’s instructions.

Class 1, 2, and 3 integrons were detected both from stools and from GNB using a multiplex qPCR, as previously described (Barraud et al., 2010). For all qPCR runs, plasmids pBAD18::*intI1*, pGEM^®^-T Easy::*intI2* and pBAD18::*intI3* as positive controls. So as to check for PCR inhibitors, a qPCR targeting the 16S DNA gene was performed (Relman, 1993). All qPCR assays were performed on the MX3005P system (Agilent^®^).

For each stool sample, all isolates belonging to a same species but with different AST and/or integron profiles were considered as different strains.

### Statistical Analysis

Mc Nemar Chi^2^ test for paired data was used to evaluate the proportions of integrons (all integrons, class 1 integrons only, class 2 integrons only and class 1 and 2) detected with the cultivation-dependent approach and the cultivation-independent approach. The association between previous or on-going antibiotherapy and integron content was tested using Chi^2^ test or Fisher exact test according to the theoretical effectives.

Link between integron content and AMR phenotypes was analyzed using the Chi^2^ test and the Fisher’s exact test for group smaller than 5, as well as to compare integron carriage results of different groups.

### Ethics Statement

For this study, an ethics review process was not needed as stool samples were obtained through standard care. The stools samples from GP patients working in non-clinical units of Limoges hospital and attending industrial medicine clinics were sampled for detection of pathogens like *Salmonella* and stool samples from ICU patients were sampled for digestive screening of multidrug-resistant bacteria carriage. All subjects were informed that their stool samples could be used for a clinical research purpose and they were able to express their opposition. We declared the human stool collections to the French “Ministère de l’Enseignement et de la Recherche” under CODECOH number DC-2008-604. Moreover, this study was approved by the scientific committee of the sponsor, Limoges hospital.

## Results

### Human and Animal Populations

Characteristics of the three populations (number of subjects, sex ratio, mean age and on-going or previous antibiotic therapy) are described in Supplementary Table S1. Antibiotic consumption was predominant in ICU patients.

### Bacterial Culture

Culture was negative for 30% of ICU stools, whereas only for 2% of GP and BOV. A total of 1012 different GNB were isolated from the 488 stools with a positive culture (Supplementary Table S2). The average number of GNB per stool was 1.9, 1.4, and 2.3 for GP, ICU, and BOV, respectively. *Escherichia coli* was the most frequent species: 67.1% of all GNB (81.1, 55.5 and 63.0% for GP, ICU, and BOV respectively). Other major species were *Citrobacter freundii* (*n* = 10) and *Klebsiella pneumoniae* (*n* = 9) for GP, *K. pneumoniae* (*n* = 32) followed by *Pseudomonas aeruginosa* (*n* = 23) and *K. oxytoca* (*n* = 22) for ICU, *E. fergusonii* (*n* = 25) and *Acinetobacter lwoffi* (*n* = 22) for BOV.

### GNB and Integron Content

Prevalence of integrons was 16.2, 23.8, and 14.2% among GNB isolated from GP, ICU, and BOV respectively, with mainly class 1 integrons (Supplementary Table S2). *E. coli* was the main species carrying integrons in the three populations with respectively 57, 52, and 36 integron-positive isolates in GP, ICU and BOV. In GP and BOV, respectively 3 and 6 GNB other than *E. coli* were integron-positive: *Proteus mirabilis* (3), *Proteus vulgaris* (1), *Providencia rettgeri* (1), *Enterobacter cloacae* (1), *Citrobacter braakii* (1), *Salmonella* Typhimurium (1) and *Acinetobacter lwoffi* (1). In ICU, 32 other GNB were integron-positive, mainly *K. pneumoniae* (*n* = 11), *K. oxytoca* (*n* = 8), and *P. aeruginosa* (*n* = 5).

Analysis of the link between integrons and antibiotic resistance was determined for *E. coli*. We observed a gradual increase in the percentage of integron-positive *E. coli* correlated with the number of acquired resistances (Supplementary Table S3). Globally, the percentage of *E. coli* with resistance to at least two antibiotics was higher among integron-positive isolates than among integron-negative isolates (95.3% versus 23.9%; *p* < 0.001). Moreover, a statistically significant link was observed between integron and acquired resistance to most antibiotics: penicillins, penicillins and inhibitors, almost all aminoglycosides (except amikacin), quinolones, sulfamethoxazole, trimethoprim, tetracycline, and chloramphenicol (Supplementary Table S4). In all three populations, negative predictive values (NPVs) of integron carriage for antibiotic resistance were up to 97% for third generation cephalosporins, up to 98% for aminoglycosides used in therapeutics (i.e., gentamicin, amikacin, and tobramycin), up to 93% for fluoroquinolones and up to 92% for SXT.

### Digestive Carriage of Integrons

With the cultivation-dependent approach based on integron detection from GNB isolates (number of patients with at least 1 GNB carrying an integron), the integron carriage of the subjects was 23.2, 26.9% for GP and ICU patients respectively and 24.2% for bovines (**Table 1**). With the cultivation-independent approach (number of patients with integron detection by qPCR directly from stools), the integron carriage was at least twofold higher in the three populations: 43.8, 52.7, and 65.6% for GP, ICU, and BOV respectively (**Table 1**). These differences between the two approaches were statistically significant for the three populations (*p* < 0.01). In humans (GP/ICU), integron carriage was mainly due to class 1 integrons, class 2 integrons being present in only 5–6% of the subjects. In bovines, integron carriage was due to both class 1 and class 2 integrons, 57.1 and 39.1% respectively. No class 3 integron was detected. We did not find any significant difference in integron carriage along the 4 years of the study (data not shown).

**Table 1:** Digestive carriage of integrons.

		Cultivation-dependent approach	Cultivation-independent approach	*p*^∗^
**GP** (*n* = 194)	**All integrons**	**45 (23.2%)**	**85 (43.8%)**	<0.01
	Class 1 integrons only	41 (21.2%)	75 (38.7%)	<0.01
	Class 2 integrons only	2 (1.0%)	6 (3.1%)	0.04
	Class 1 and 2 integrons	2 (1.0%)	4 (2.1%)	0.41
**ICU** (*n* = 245)	**All integrons**	**66 (26.9%)**	**129 (52.7%)**	<0.01
	Class 1 integrons only	63 (25.7%)	114 (46.5%)	<0.01
	Class 2 integrons only	1 (0.4%)	7 (2.9%)	0.03
	Class 1 and 2 integrons	2 (0.8%)	8 (3.3%)	0.06
**BOV** (*n* = 128)	**All integrons**	**31 (24.2%)**	**84 (65.6%)**	<0.01
	Class 1 integrons only	27 (21.1%)	34 (26.6%)	0.25
	Class 2 integrons only	3 (2.3%)	11 (8.6%)	0.03
	Class 1 and 2 integrons	1 (0.8%)	39 (30.5%)	<0.01

*Numbers indicate subjects being integron carriers among each population. Class 3 integrons were not detected. ^∗^Test used was the Mc Nemar Chi^*2*^ test for paired data.*

### Concordance between the Two Approaches

Results between the cultivation-dependent and the cultivation-independent approaches were compared for all the 567 samples. Results were concordant for 383 samples (67.5%) with 255 being integron-negative and 128 integron-positive with the two approaches. For the remaining samples, the cultivation-independent approach allowed the detection of integrons in 170 samples for whom the culture did not find integron-positive isolates. On the other hand, for 14 patients, we detected integrons with the cultivation-dependent approach and not with the cultivation-independent approach.

### Integron Carriage and Antibiotic Selection Pressure

Differences of integron carriage between the two human populations (GP and ICU) were not significant whatever the approach (*p* = 0.37 with data from the cultivation-dependent approach; *p* = 0.065 with data from the cultivation-independent approach). The integron carriage was also not significantly different between GP and ICU populations according to the antibiotic consumption, whatever the approach (**Tables 2A,B**).

**Table 2:** Integron digestive carriage and antibiotic therapy with data from the cultivation-dependent approach.

(A)			
GP^∗^ (*n* = 188)			
	Integron-positive	Integron-negative	*p*
No antibiotic therapy	43 (24.9%)	130 (75.1%)	0.32¤
Previous or on-going antibiotic therapy	2 (13.3%)	13 (86.7%)	

**ICU (*n* = 245)**			

	Integron-positive	Integron-negative	*p*
No antibiotic therapy	9 (25.0%)	27 (75.0%)	0.78^#^
Previous or on-going antibiotic therapy	57 (27.3%)	152 (72.7%)	

**(B)**			
**GP^∗^ (*n* = 188)**			

	Integron-positive	Integron-negative	*p*
No antibiotic therapy	77 (55.5%)	96 (44.5%)	0.40^#^
Previous or on-going antibiotic therapy	5 (66.7%)	10 (33.3%)	

**ICU (*n* = 245)**			

	Integron-positive	Integron-negative	*p*
No antibiotic therapy	23 (36.1%)	13 (63.9%)	0.14^#^
Previous or on-going antibiotic therapy	106 (50.7%)	103 (49.3%)	

*Numbers indicate subjects being integron-positive or integron-negative carriers. ^∗^Patients with no data for antibiotic therapy were excluded. Test used was the Chi^2^ test^#^ or the Fisher’s exact test¤.*

## Discussion

In this study based on a “One Health” approach, we have retrospectively taken benefit from a stool collection from human and bovine subjects living in a cattle-rearing area in France, to check for their digestive carriage of integrons. Indeed, integrons are now well-recognized as genetic biomarkers of acquired resistance (Leverstein-van Hall et al., 2003; Barraud et al., 2014) and they can provide a reliable estimation of AMR dissemination (Amos et al., 2015; Gillings et al., 2015). The “One Health” approach is now required to tackle AMR, human and animal health being interconnected, and international declarations on AMR pay particular attention to this aspect in action plans.

In our work, the integron carriage was studied with both methods: a cultivation-dependent approach based on GNB, as classically determined (Skurnik et al., 2005, 2008; Vasilakopoulou et al., 2009; Bailey et al., 2010; Kheiri and Akhtari, 2016) and a cultivation-independent approach with a qPCR directly applied from stool DNA. This cultivation-independent approach is novel and complementary to the classical approach. To our knowledge, such an approach was previously used only once for determination of the integron carriage in cattle, not directly from stools but from an enrichment broth (Barlow et al., 2004). Even though their methodology was slightly different than ours, the authors observed integron carriage rates from 50% for class 1 integrons and 28% for class 2 integrons with the cultivation-dependent approach to 86 and 94% using a direct detection of integrons from stool DNA after an enrichment step. In our study, we also observed an increase of the integron carriage with the cultivation-independent approach, with at least a twofold increase (**Table 1**) and the cultivation-independent approach allowed detecting 170 integron carriers that were considered as non-carriers with the cultivation-dependent approach. This increase was expected. First, some bacteria may have been not collected during the culture process: (i) GNB colonies were visually selected and some strains may have been missed, notably if morphotypes were similar; (ii) culture of one integron-positive GNB could have been not observed due to competition with other more predominant GNB; (iii) not all GNB were able to grow on Drigalski agar plates, like *Campylobacter* or *Pasteurella* (Lee et al., 2002; Kehrenberg and Schwarz, 2011). Moreover, it has been shown that Gram-positive bacteria, especially Corynebacteria, may contain integrons (Nesvera et al., 1998; Nandi et al., 2004; Barraud et al., 2011). However, our method using Drigalski agar plates only allowed isolating GNB. Using the cultivation-independent method, these Gram-positive bacteria containing integrons can be detected. Lastly, bacteria from patients under antibiotics may have been unable to grow. This is in agreement with the fact that in ICU patients exposed to a high antibiotic selective pressure, culture was negative for 30% of stools. Lastly, we know that culturing allows detection of a very limited part of all the species present in the gut microbiota (Harmsen and de Goffau, 2016) and we can expect that non-cultivable bacteria can also contain integron. All these explanations argue that the cultivation-independent approach would constitute a complementary approach for the determination of the digestive carriage of integrons than cultivation-dependent approaches, notably when an antibiotic treatment is ongoing. The benefit of cultivation-independent approaches for the diagnostic of antibiotic resistance has been previously shown (Decousser et al., 2017; Dunne et al., 2017). However, for 14 subjects, a signal was positive *via* the cultivation-dependent approach but negative *via* the cultivation-independent approach. Hypothesis of inhibitors from stools was excluded thanks to correct amplification of the 16S-DNA from all the samples. For these patients, the number of integron-positive strains was probably very low, under the threshold of detection by qPCR. The culture method with an enrichment broth allowed to isolate these bacteria. This confirms that both cultivation-dependent and cultivation-independent approach are complementary.

For humans, independently of the method used, integron carriage was mainly due to class 1 integrons (79 out of 85 integron carriers for GP, and 122 out of 129 for ICU), class 2 being present in only 11% of the integron carriers. This is in agreement with previous studies on integron detection in commensal *E. coli* (Bailey et al., 2010; Copur-Cicek et al., 2014; Kheiri and Akhtari, 2016) or in clinical isolates (Barraud et al., 2014; Malek et al., 2015; Xu et al., 2017). For bovines, results were different from humans, notably when looking at the cultivation-independent approach. Even if class 1 integrons remained the most prevalent (73 out of the 84 carriers), class 2 integrons were detected among 50 out of the 84 bovine integron carriers. These results were not obtained when looking at the culture with only 2 out of the 36 bovine carriers being positive for class 2 integrons. This discrepancy was also retrieved by Barlow et al. (2004) who obtained 94% of cattle fecal samples positive for class 2 integrons from enrichment broths *versus* 28% from GNB. Other studies indicated that class 2 integrons seemed more related to animals than to humans (Lapierre et al., 2008; Barlow et al., 2009). One can hypothesize that bacteria from the bovine microbiota constitute a yet unknown reservoir for class 2 integrons. Animal and human gut constitute a huge reservoir of resistances genes (Fitzpatrick and Walsh, 2016). If animals are reservoirs for class 2 integrons, one can expect that presence of such a class within humans may be related to contacts with animals or via the food chain. It is noteworthy that no class 3 integron was detected even using the cultivation-independent approach. It has been previously hypothesized that class 3 integrons are mainly distributed in the environment (Stalder et al., 2014; Simo Tchuinte et al., 2016), and exceptionally within humans or animals.

Rates of integron carriage in human obtained with the cultivation-dependent approach are similar to data found in previous studies, between 15 and 30% (Leverstein-Van Hall et al., 2002; Vinue et al., 2008; Vasilakopoulou et al., 2009). In previous studies, antibiotic therapy was identified as a risk factor for integron acquisition, both in humans (Nijssen et al., 2005; Skurnik et al., 2005; van der Veen et al., 2009) and in animals (Barlow et al., 2009; Wu et al., 2011). Skurnik et al. (2005, 2008) first reported different *E. coli* integron carriage in animals according to their degrees of exposure to humans. Authors observed a gradual increase in the prevalence of class 1 integrons correlated with the increase in exposure to human. In our study, antibiotic selection pressure was very different between GP and ICU with 8% of GP subjects exposed to antibiotics *versus* 83% of ICU patients. However, in our study, we did not find statistical difference in integron carriage between GP and ICU, whatever the approach.

*Escherichia coli* was the predominant species in all three populations. A significant link was observed in *E. coli* between presence of integrons and acquired resistance to at least two antibiotics (*p* < 0.001). This was demonstrated previously whatever the definition of the multiresistance (Martinez-Freijo et al., 1998; Leverstein-van Hall et al., 2003; Shahi and Kumar, 2015; Ochoa et al., 2016). Here, we observed that when an integron was present in an *E. coli* isolate, the most frequent phenotype was the association of a low level penicillinase conferring resistance to amoxicillin and ticarcillin with co-resistances to trimethoprim, sulfamethoxazole and tetracycline (data not shown). As we showed recently (Barraud et al., 2014), integrons alone clearly cannot explain the resistance to all of the tested antibiotics, but integrons are usually carried by plasmids or transposons, which can also host other resistance genes. It has been shown in humans and animals that *E. coli* can harbor plasmids with different genes encoding these resistances (to ampicillin, tetracycline, trimethoprim, and sulfonamides) and that some of these plasmids often carry integrons (Kirchner et al., 2014; Jackson et al., 2015). A statistically positive significant link was also observed between integrons and each antibiotic, except for carbapenems, third generation cephalosporins and amikacin. Finally, NPVs – i.e., when an integron is absent, the strain has a high probability to be susceptible – were >97% for third generation cephalosporins, up to 98% for aminoglycosides used in therapeutics, up to 93% for fluoroquinolones and up to 92% for SXT. This confirms our previous data aiming at considering integrons as relevant biomarkers of antibiotic resistance with a good NPV (Barraud et al., 2014). On the other hand, most positive predictive values (PPV) – i.e., when an integron is present, the strain has a high probability to be resistant – were below 95% or even lower for all the antibiotics (Supplementary Table S3). This may be explained by the low prevalence of multi-drug resistant enterobacteria in our county.

Our study aimed at determining the digestive carriage of integrons among human and bovine populations. We showed that the cultivation-independent approach increased significantly, by twofold, the rates of integron carriers compared to the cultivation-dependent approach. It is likely that non-cultivable bacteria of the gut harbor integrons. This high frequency of carriage of integrons in the gut is of clinical significance. Indeed, integrons are known to be very efficient genetic elements for capture, expression and dissemination of antibiotic resistance gene cassettes and they can harbor a wide diversity of gene cassettes encoding resistance to almost all antibiotic families. Antibiotics are known to alter the gut microbiota and lead to selection and emergence of resistant bacteria (Ianiro et al., 2016). Furthermore, it is known that antibiotics induce the mechanism of acquisition of antibiotic resistance gene cassettes by integrons (Guerin et al., 2009). Our findings thus highlight that the gut is a reservoir for integrons that can easily acquire and exchange resistant genes under antibiotic selective pressure leading to the dissemination of resistant bacteria.

Here, we showed that both cultivation-dependent and -independent methods are complementary for the detection of integrons. However, although the cultivation-independent approach gives new insights about the epidemiology of integrons, we advocate for the maintenance of the cultivation-dependent approach that is the sole approach able to provide, at the species level, the link between integrons and antibiotic resistance. This study also highlights that the gut, in both humans and animals, is a high potential reservoir of genetic determinants for antibiotic resistance, that can be further acquired by pathogens.

## Author Contributions

OB performed analysis, wrote the manuscript; DC performed analysis, wrote the manuscript; GM performed analysis; EC-D reviewed the manuscript; BF enrolled ICU patients, reviewed the manuscript; C-YC collected animal stool samples; M-CP designed the study, wrote and reviewed the manuscript.

## Conflict of Interest Statement

The authors declare that the research was conducted in the absence of any commercial or financial relationships that could be construed as a potential conflict of interest.
