# Supplementary material for: Integron Digestive Carriage in Human and Cattle: A “One Health” Cultivation-Independent Approach
Source: Front Microbiol. 2017 Sep 27;8:1891. doi: 10.3389/fmicb.2017.01891 (PMC5624303; doi:10.3389/fmicb.2017.01891)
Supplement: Supplementary file 1 [file Table_1.docx]

Table S1: Flow chart of the population

|  | **GP** | **ICU** | **BOV** |
| --- | --- | --- | --- |
| Number of subjects | 194 | 245 | 128 |
| Origin | Subjects working in nonclinical units of Limoges hospital and attending industrial medicine clinics | ICU patients from Limoges hospital | Bovines from 55 different farms |
| Sex ratio | 0.69 | 1.72 | - |
| Mean age | 37.9 (18-60) | 61.4 (18-94) | ND |
| On-going and previous antibiotic therapy in the past 3 months | No: 89.2%  Yes: 7.7%  Unknown: 3.1% | No: 17.1%  Yes: 82.9% | ND |

ND: not determined
