# Supplementary material for: Integron Digestive Carriage in Human and Cattle: A “One Health” Cultivation-Independent Approach
Source: Front Microbiol. 2017 Sep 27;8:1891. doi: 10.3389/fmicb.2017.01891 (PMC5624303; doi:10.3389/fmicb.2017.01891)
Supplement: Supplementary file 2 [file Table_2.docx]

Table S2: Bacterial cultures.

|  | **GP** | **ICU** | **BOV** |  |
| --- | --- | --- | --- | --- |
| Number of GNB | 370 | 353 | 289 |  |
| Integron-positive GNB (%)   - class 1 integrons - class 2 integrons - class 1 and class 2 integrons - class 3 integrons | | **60 (16.2%)**  56 (15.1%)  3 (0.8%)  1 (0.3%)  0 | **84 (23.8%)**  78 (22.1%)  6 (1.7%)  0  0 | **41 (14.2%)**  37 (12.8%)  4 (1.4%)  0  0 |
| Number of *Escherichia coli* | 300 | 197 | 182 |  |
| Integron-positive *E. coli* (%)   - class 1 integrons - class 2 integrons - class 1 and class 2 integrons - class 3 integrons | **57 (19.0%)**  53 (17.7%)  3 (1.0%)  1 (0.3%)  0 | **52 (26.4%)**  50 (25.4%)  2 (1.0%)  0  0 | **36 (19.8%)**  34 (18.7%)  2 (1.1%)  0  0 |  |
