# Supplementary material for: Integron Digestive Carriage in Human and Cattle: A “One Health” Cultivation-Independent Approach
Source: Front Microbiol. 2017 Sep 27;8:1891. doi: 10.3389/fmicb.2017.01891 (PMC5624303; doi:10.3389/fmicb.2017.01891)
Supplement: Supplementary file 3 [file Table_3.docx]

Table S3: Link between integrons and multidrug resistance among *E. coli*.

| Number of resistances | 0-2 | 3-4 | 5-6 | >6 |
| --- | --- | --- | --- | --- |
| Integron-negative *E. coli* (%) | 98.3 | 82.0 | 53.1 | 30.2 |
| Integron-positive *E. coli* (%) | 1.7 | 18.0 | 46.9 | 69.8 |
