# Supplementary material for: Integron Digestive Carriage in Human and Cattle: A “One Health” Cultivation-Independent Approach
Source: Front Microbiol. 2017 Sep 27;8:1891. doi: 10.3389/fmicb.2017.01891 (PMC5624303; doi:10.3389/fmicb.2017.01891)
Supplement: Supplementary file 4 [file Table_4.docx]

Table S4: Link between integrons and antibiotic resistance among *Escherichia coli*.

|  | **GP**  *E. coli* (n=300) / integron-positive *E. coli* (n=57) | | | | | |  | **ICU**  *E. coli* (n=197) / integron-positive *E. coli* (n=52) | | | | | |  | **BOV**  *E. coli* (n=182) / integron-positive *E. coli* (n=36) | | | | | |
| --- | --- | --- | --- | --- | --- | --- | --- | --- | --- | --- | --- | --- | --- | --- | --- | --- | --- | --- | --- | --- |
|  | Percentage of resistant isolates (%) | | | Predictive values | | |  | Percentage of resistant isolates (%) | | | Predictive values | | |  | Percentage of resistant isolates (%) | | | Predictive values | | |
|  | All | *intI*- | *intI*+ | *p* | NPV | PPV |  | all | *intI*- | *intI*+ | *p* | NPV | PPV |  | all | *intI*- | *intI*+ | *p* | NPV | PPV |
| AMX | 34.7 | 25.8 | 71.9 | <0.01^¤^ | 74.2 | 71.9 |  | 42.1 | 26.9 | 84.6 | <0.01^#^ | 73.1 | 84.6 |  | 31.3 | 16.4 | 91.7 | <0.01^#^ | 83.6 | 91.7 |
| AMC | 13.7 | 10.2 | 28.1 | <0.01^#^ | 89.8 | 28.1 |  | 17.3 | 10.3 | 36.5 | <0.01^#^ | 89.7 | 36.5 |  | 19.8 | 7.5 | 69.4 | <0.01^#^ | 92.5 | 69.4 |
| TIC | 31.3 | 21.7 | 71.9 | <0.01^#^ | 78.3 | 71.9 |  | 40.1 | 24.8 | 82.7 | <0.01^#^ | 75.2 | 82.7 |  | 29.1 | 14.4 | 88.9 | <0.01^#^ | 85.6 | 88.9 |
| TCC | 10.0 | 7.8 | 19.3 | <0.01^#^ | 92.2 | 19.3 |  | 16.8 | 9.7 | 36.5 | <0.01^#^ | 90.3 | 36.5 |  | 20.3 | 7.5 | 72.2 | <0.01^#^ | 92.5 | 72.2 |
| CTX | 1.3 | 0.8 | 3.5 | 0.16^¤^ | 99.2 | 3.5 |  | 1.5 | 1.4 | 1.9 | 1.00^¤^ | 98.6 | 1.9 |  | 0.5 | 0.0 | 2.8 | 0.19^¤^ | 100 | 2.8 |
| CAZ | 0.3 | 0.0 | 1.8 | 0.19^¤^ | 100 | 1.8 |  | 2.5 | 2.1 | 3.8 | 0.61^¤^ | 97.9 | 3.8 |  | 2.2 | 0.7 | 8.3 | 0.02^¤^ | 99.3 | 8.3 |
| IPM | 0.0 | 0.0 | 0.0 | - | 100 | 0.0 |  | 0.0 | 0.0 | 0.0 | - | 100 | 0.0 |  | 0.0 | 0.0 | 0.0 | - | 100 | 0.0 |
| K | 9.7 | 7.8 | 17.5 | 0.02^#^ | 92.2 | 17.5 |  | 10.7 | 6.9 | 21.2 | <0.01^#^ | 93.1 | 21.2 |  | 16.5 | 9.6 | 44.4 | <0.01^#^ | 90.4 | 44.4 |
| GM | 0.3 | 0.0 | 1.8 | 0.19^¤^ | 100 | 1.8 |  | 3.0 | 0.7 | 9.6 | <0.01^¤^ | 99.3 | 9.6 |  | 8.2 | 1.4 | 36.1 | <0.01^¤^ | 98.6 | 36.1 |
| TM | 2.7 | 1.2 | 8.8 | 0.01^¤^ | 98.8 | 8.8 |  | 5.1 | 2.1 | 13.5 | <0.01^¤^ | 97.9 | 13.5 |  | 9.3 | 1.4 | 41.7 | <0.01^¤^ | 98.6 | 41.7 |
| AN | 1.0 | 0.0 | 5.3 | 0.01^¤^ | 100 | 5.3 |  | 0.0 | 0.0 | 0.0 | - | 100 | 0.0 |  | 0.5 | 0.0 | 2.8 | 0.20^¤^ | 100 | 2.8 |
| S | 30.3 | 18.4 | 80.7 | <0.01^#^ | 81.6 | 80.7 |  | 31.5 | 19.3 | 65.4 | <0.01^#^ | 80.7 | 65.4 |  | 47.3 | 35.6 | 94.4 | <0.01^#^ | 64.4 | 94.4 |
| SPT | 10.7 | 2.5 | 45.6 | <0.01^#^ | 97.5 | 45.6 |  | 9.6 | 1.4 | 32.7 | <0.01^#^ | 98.6 | 32.7 |  | 19.8 | 6.2 | 75.0 | <0.01^#^ | 93.8 | 75.0 |
| NA | 4.3 | 2.1 | 14.0 | <0.01^¤^ | 97.9 | 14.0 |  | 14.2 | 10.3 | 25.0 | <0.01^#^ | 89.7 | 25.0 |  | 17.6 | 5.5 | 66.7 | <0.01^#^ | 94.5 | 66.7 |
| PEF | 1.7 | 0.0 | 8.8 | <0.01^¤^ | 100 | 8.8 |  | 10.2 | 6.2 | 21.2 | <0.01^#^ | 93.8 | 21.2 |  | 14.8 | 4.1 | 58.3 | <0.01^#^ | 95.9 | 58.3 |
| CIP | 1.7 | 0.0 | 8.8 | <0.01^¤^ | 100 | 8.8 |  | 10.2 | 6.2 | 21.2 | <0.01^#^ | 93.8 | 21.2 |  | 13.2 | 3.4 | 52.8 | <0.01^#^ | 96.6 | 52.8 |
| SSS | 26.7 | 15.2 | 75.4 | <0.01^#^ | 84.8 | 75.4 |  | 32.5 | 13.8 | 84.6 | <0.01^#^ | 86.2 | 84.6 |  | 33.5 | 18.5 | 94.4 | <0.01^#^ | 81.5 | 94.4 |
| TMP | 15.3 | 1.6 | 73.7 | <0.01^#^ | 98.4 | 73.7 |  | 23.9 | 4.8 | 76.9 | <0.01^#^ | 95.2 | 76.9 |  | 18.1 | 4.8 | 72.2 | <0.01^#^ | 95.2 | 72.2 |
| SXT | 18.3 | 5.3 | 73.7 | <0.01^#^ | 94.7 | 73.7 |  | 24.4 | 6.9 | 73.1 | <0.01^#^ | 93.1 | 73.1 |  | 20.9 | 7.5 | 75.0 | <0.01^#^ | 92.5 | 75.0 |
| C | 13.0 | 8.6 | 31.6 | <0.01^#^ | 91.4 | 31.6 |  | 15.2 | 9.0 | 32.7 | <0.01^#^ | 91.0 | 32.7 |  | 24.2 | 11.0 | 77.8 | <0.01^#^ | 89.0 | 77.8 |
| TE | 32.7 | 24.6 | 66.7 | <0.01^#^ | 75.4 | 66.7 |  | 34.5 | 20.7 | 73.1 | <0.01^#^ | 79.3 | 73.1 |  | 33.0 | 20.5 | 83.3 | <0.01^#^ | 79.5 | 83.3 |

AMX: amoxicillin, AMC: amoxicillin/clavulanic acid, TIC: ticarcillin, TCC: ticarcillin/clavulanic acid, CTX: cefotaxime, CAZ: ceftazidime, IPM: imipenem, K: kanamycin, GM: gentamicn, TM: tobramycin, AN: Amikacin, S: streptomycin, SPT: spectinomycin, NA: nalidixic acid, PEF: pefloxacin, CIP: ciprofloxacin, SSS: sulfamethoxazole, TMP: trimethoprim, SXT: trimethoprim/sulfamethoxazole, C: chloramphenicol, TE : tetracyclin. PPV: Positive predictive value. NPV: Negative predictive value. ^#^Test used were the chi2 test. ^¤^Test used were the Fisher’s exact test.
